# Condemned or Not to Die? Gene Polymorphisms Associated With Cell Death in Pemphigus Foliaceus

**DOI:** 10.3389/fimmu.2019.02416

**Published:** 2019-10-18

**Authors:** Valéria Bumiller-Bini, Gabriel Adelman Cipolla, Mariana Basso Spadoni, Danillo Gardenal Augusto, Maria Luiza Petzl-Erler, Marcia Holsbach Beltrame, Angelica Beate Winter Boldt

**Affiliations:** ^1^Laboratory of Human Molecular Genetics, Department of Genetics, Federal University of Paraná, Curitiba, Brazil; ^2^Department of Neurology, University of California, San Francisco, San Francisco, CA, United States

**Keywords:** pemphigus, cell death, skin disease, autoimmune disease, apoptosis, pyroptosis, necroptosis, immunogenic cell death

## Abstract

Pemphigus foliaceus (PF) is an autoimmune blistering skin disease that occurs sporadically across the globe and is endemic in Brazil. Keratinocyte adhesion loss (acantholysis) is associated with high levels of anti-desmoglein 1 IgG autoantibodies, but the role of cell death is poorly understood in PF. Current evidence disqualifies apoptosis as the major cell death mechanism and no other process has yet been investigated. To approach the role of variation in genes responsible for cell death pathways in pemphigus susceptibility, we systematically investigated the frequencies of 1,167 polymorphisms from genes encoding products of all 12 well-established cell death cascades (intrinsic and extrinsic apoptosis, necrosis, necroptosis, ferroptosis, pyroptosis, parthanatos, entotic, NETotic, lysosome-dependent, autophagy-dependent, and immunogenic). By multivariate logistic regression, we compared allelic and genotypic frequencies of 227 PF patients and 194 controls obtained by microarray hybridization. We found 10 variants associated with PF (*p* < 0.005), belonging to six cell death pathways: apoptosis (*TNF, TRAF2, CD36*, and *PAK2*), immunogenic cell death (*EIF2AK3, CD47*, and *SIRPA*), necroptosis (*TNF* and *TRAF2*), necrosis (*RAPGEF3*), parthanatos (*HK1*), and pyroptosis (*PRKN*). Five polymorphisms were associated with susceptibility: TNF rs1800630*A (*OR* = 1.9, *p* = 0.0003), CD36 rs4112274*T (*OR* = 2.14, *p* = 0.0015), CD47 rs12695175*G (*OR* = 1.77, *p* = 0.0043), SIRPA rs6075340*A/A (*OR* = 2.75, *p* = 0.0009), and HK1 rs7072268*T (*OR* = 1.48, *p* = 0.0045). Other five variants were associated with protection: TRAF2 rs10781522*G (*OR* = 0.64, *p* = 0.0014), PAK2 rs9325377*A/A (*OR* = 0.48, *p* = 0.0023), EIF2AK3 rs10167879*T (*OR* = 0.48, *p* = 0.0007), RAPGEF3 rs10747521*A/A (*OR* = 0.42, *p* = 0.0040), and PRKN rs9355950*C (*OR* = 0.57, *p* = 0.0004). Through functional annotation, we found that all associated alleles, with the exception of PRKN rs9355950*C, were previously associated with differential gene expression levels in healthy individuals (mostly in skin and peripheral blood). Further functional validation of these genetic associations may contribute to the understanding of PF etiology and to the development of new drugs and therapeutic regimens for the disease.

## Introduction

Pemphigus foliaceus (PF) is an autoimmune bullous disease of the skin, characterized by the production of autoantibodies that recognize the desmosome protein desmoglein 1 (DSG1) (1, 2). The binding of antibodies to this cell adhesion molecule is accompanied by acantholysis (keratinocyte detachment) and lesions in the superficial granular layer of the epidermis. In contrast, a different form of pemphigus called pemphigus vulgaris (PV) is characterized by anti-desmoglein 3 antibodies, which bind in deeper layers of the skin and cause blisters in the skin and mucosa (3). PF occurs sporadically around the world−0.75–5 cases/million per year (4). In Brazil, PF is endemic and commonly known as *fogo selvagem* (“wild fire” in Portuguese). The prevalence of PF in Limão Verde, located in the Brazilian state of Mato Grosso do Sul, is of 3.04%, one of the highest prevalences ever reported for autoimmune diseases (5). PF pathogenesis is multifactorial, resulting from poorly understood interactions between multiple environmental and genetic factors (6, 7).

### Cell Death in Pemphigus: An Unsolved Issue

Despite the pathogenic relevance of IgG autoantibodies in the acantholytic process, the exact mechanisms that lead keratinocytes to death remain unknown (8). Apoptosis has been suggested to play an important role in some dermatoses with positive Nikolsky's sign (skin detachment after slight rubbing) as in PV and PF (9–12), either prior (10, 13–15), or after the loss of cell adhesion (8, 16–18). As early as 1998, Gniadecki et al. reported many apoptotic keratinocytes in acantholytic tissue and in the cohesive epidermis just under the blisters of sporadic PF and PV lesional skin biopsies, as judged by positive TUNEL signs (terminal deoxynucleotidyl transferase dUTP nick end labeling) (9). Rodrigues et al. (19) also found TUNEL-positive keratinocytes in 12/13 biopsies of perilesional skin of endemic PF lesions. Among them, 10/13 presented keratinocytes with intense expression of proapoptotic inducible nitric oxide synthase (iNOS) and 3/13, with rather discrete-moderate expression of proapoptotic FAS protein. Anti-apoptotic Bcl-2 occurred in 4/13 biopsies only, being much more abundant in the inflammatory infiltrate, which also had discrete-moderate expression of interleukin 1, interferon gamma, and tumor necrosis factor alpha (TNF-α) proinflammatory cytokines (11/13) (19). After the passive transfer of sporadic PF-antibodies in the experimental neonatal mouse model, keratinocytes expressed the proapoptotic Bax factor, followed by activation of caspases (CASP) 3 and 6, and down-regulation of the anti-apoptotic Bcl-x(L) factor. In this model, apoptotic inhibitors abrogated the acantholytic process (14). Nevertheless, p38MAPK signaling occurred in this same model in two phases, and the first peak of activation coincided with acantholysis, prior to the second peak that induced activation of CASP-3 (18). Taking into account ultra-violet (UV) light exposure as a known risk factor for endemic PF, it is interesting that caspases-3 and -7 cleave desmoglein-1 intracellularly and that knock-down of desmoglein-1 protects cells from UV induced apoptosis in irradiated keratinocytes (20).

On the other hand, only few apoptotic cells were detected in skin biopsies of endemic PF patients, whereas p63 marked many undifferentiated cells distributed over the whole epidermis, both in injured and non-injured skin (21). Electron microscopy did not reveal any morphological signs of apoptosis—retraction of pseudopods, pyknosis, karyorrhexis, and plasma membrane blebbing—in acantholytic tissue of PV and PF patients (8, 22). There were, as well, no signs for activation of caspases (as cleaved CASP3 and CASP8, fractin, or nuclear poly (ADP-ribose) polymerase—PARP) in PV and PF biopsies, nor in PV or PF IgG–incubated healthy breast reduction skin biopsies (8). A possibility suggested by Schmidt and Waschke (16) is that caspase signaling adds in desmosome destabilization, but that apoptosis itself is not responsible nor needed for acantholysis to occur.

Thus, whereas some authors state that apoptosis causes cell death in PF (9, 19, 23), others found no clear evidence of such event (8, 17, 18, 22). The uncertainty about how cell death takes place in PF, as well as the scarcity of genetic association studies on this topic, prompted the current investigation encompassing genes of all known cell death routes. In fact, there are several pathways orchestrating cell death, classified following morphological, biochemical, and functional features. In 2018, the Nomenclature Committee on Cell Death (NCCD) proposed 12 pathways orchestrating cell death, supported by genetic, biochemical, and functional results: intrinsic apoptosis, extrinsic apoptosis, mitochondrial permeability transition (MPT)-driven necrosis, necroptosis, ferroptosis, pyroptosis, parthanatos, entotic, NETotic, lysosome-dependent, autophagy-dependent, and immunogenic pathways (24). All of them are classified as regulated cell death (RCD) routes. All routes depend on the molecular machinery (causing the activation of one or more signal transduction pathways), which can be pharmacologically and/or genetically modulated. RCD begins with excessive cellular stress, caused by non-coped perturbations of the intra- and extracellular environment (24, 25). Given the poorly understood underlying mechanisms leading to keratinocyte death in PF, we intended to identify genetic variants associated with PF analyzing variants from genes whose products are known to be directly involved in RCD routes.

## Materials and Methods

### Population Sample

A total of 227 PF patients and 194 unrelated controls with no diagnosis or familial history of autoimmune illnesses were analyzed in this study. Patients received clinical and/or immunohistological diagnosis. Patients and controls were recruited from 1984 to 2015 in South/Southeastern/Central-Western Brazilian hospitals: Hospital Adventista do Pênfigo (Campo Grande, Mato Grosso do Sul), Lar da Caridade—Hospital do Fogo Selvagem (Uberaba, Minas Gerais), Hospital das Clínicas—University of São Paulo (Ribeirão Preto, São Paulo), Hospital de Clínicas—Federal University of Paraná, Hospital de Dermatologia Sanitária São Roque, and Hospital Santa Casa de Misericórdia (Curitiba, Paraná). All individuals enrolled in this study were unrelated, predominantly of European ancestry and living in rural endemic areas. No history of other autoimmune diseases was reported for patients, as well as no history of any autoimmune disease for the controls. The median age was 40.9 years for patients (minimum 6, maximum 83) and 44.8 years for controls (minimum 11, maximum 86). In both groups, 52% were women. Peripheral blood was collected, from which DNA was extracted by the phenol-chloroform-isoamyl alcohol protocol (26). Patients and controls voluntarily agreed to participate in the study and gave their informed consent. This study was carried out according to the Declaration of Helsinki, with the approval from the Brazilian National Ethics Committee (CONEP) under the protocol number CAAE 02727412.4.0000.0096 and approval number 505.988.

### Selection of Candidate Genes and Genotyping

In agreement with NCCD, we selected genes encoding proteins involved in essential aspects of at least one of the following cell death cascade processes: intrinsic apoptosis, extrinsic apoptosis, mitochondrial permeability transition (MPT)-driven necrosis, necroptosis, ferroptosis, pyroptosis, parthanatos, entotic, NETotic, lysosome-dependent, autophagy-dependent, and immunogenic pathways (24). We identified the genomic positions of each gene, considering regulatory regions of one thousand base pairs upstream and downstream from the transcription start and end sites, respectively, of the longest transcript, according to the GRCh37/hg19 human genome version (available at: http://www.lgmh.ufpr.br/data/Supplementary_material_1_Bumiller-Bini_2019.xlsx).

**Figure 1:**
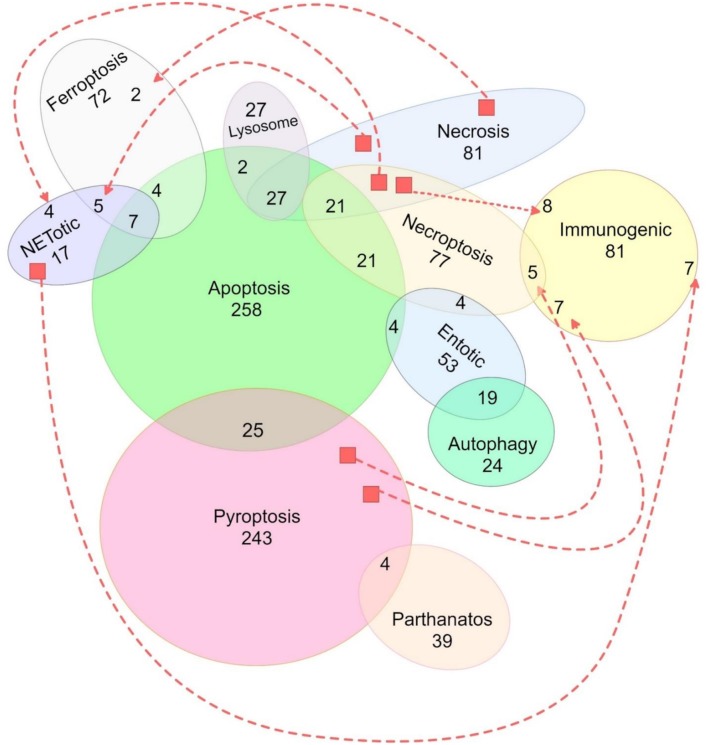
Representation of the distribution of all 1,167 SNPs used in this study according to their related cell death pathway(s). Traced lines were used to represent additional shared SNPs (unions) between two pathways that were not otherwise graphically united. Genes from the intrinsic and extrinsic apoptosis pathways were represented together. Unrepresented SNPs, shared among pathways: one between apoptosis, immunogenic, lysosome, and necrosis pathways; two between apoptosis, immunogenic, lysosome, and pyroptosis pathways; two between necroptosis, necrosis, pyroptosis, NETotic, and immunogenic pathways; three between pyroptosis and necroptosis; three between apoptosis, necroptosis, necrosis, and NETotic pathways; four between apoptosis, necrosis, and immunogenic pathways; and four between apoptosis, entotic, and immunogenic pathways).

Genotyping was performed using microarray hybridization (CoreExome-24 v1.1 Illumina®) in 194 and 227 DNA samples from controls and endemic PF patients, respectively. A total of 3,798 SNPs were extracted from DNA microarray data, filtered based on genotyping quality, and on population genetics criteria: excluding those SNPs with minor allele frequencies <1%, with genotypic distributions deviating from those expected by Hardy-Weinberg equilibrium in controls (*p* < 0.05) and/or with high linkage disequilibrium (*r*^2^ ≥ 0.8). After filtering, a total of 1,167 SNPs remained for subsequent analyses (Figure 1, set available at: http://www.lgmh.ufpr.br/data/Supplementary_material_2_Bumiller-Bini_2019.xlsx).

### Association Analysis

Association analysis was carried out by binary logistic regression, using sex and two principal components (PCA) as co-variables. The PCA eliminates spurious associations due to possible population stratification. The significance level was set to *p* < 0.005 (27). The analyses were performed using PLINK software version 1.1.9 (28). We applied Fisher's exact test to perform the stratified association analysis (29), in this case, with a significance level set to *p* < 0.05.

### *In silico* Analysis

We performed *in silico* analysis to explore the genetic associations identified in this study. Linkage disequilibrium within the Iberian population (major ancestral of the Euro-Brazilian population) was evaluated with LDlink (30). The predicted regulatory impact of the genetic variants was verified using ENSEMBL (31), GTEx portal (32), UCSC (https://genome.ucsc.edu/cgi-bin/hgGateway) (33), HaploReg (https://pubs.broadinstitute.org/mammals/haploreg/haploreg.php) (34), Innatedb (https://www.innatedb.com) (35), and Blood eQTL (expression quantitative trait loci) browser (36), which assemble public datasets, and published data. GTEx portal and the Blood eQTL browser inform whether a SNP is an expression quantitative trait loci (eQTL). Innatedb informs whether there is a physical interaction between proteins.

## Results

### Association Analysis

We found 10 SNPs associated with PF (*p* < 0.005) located in 10 different genes participating in six RCD routes: apoptosis (*CD36, PAK2, TNF*, and *TRAF2*), immunogenic cell death (*CD47, EIF2AK3*, and *SIRPA*), necroptosis (*TNF* and *TRAF2*), necrosis (*RAPGEF3*), parthanatos (*HK1*), and pyroptosis (*PRKN*) (Table 1). Altogether, the less frequent alleles of five SNPs were associated with PF susceptibility, while the less frequent alleles from the other five SNPs, with protection. All associated variants were located within non-protein coding regions.

**Table 1:** Cell death-related gene variants associated with PF.

**Gene**	**SNP**	**MAF (%)**		**Model**	**CONTR**	**CASE**	**OR**	**95%CI**	***p***
		**Contr**	**Case**						
**APOPTOSIS AND NECROPTOSIS**									
*TNF*	rs1800630	15.7	26.2	**add**	**61/327**	**117/329**	**1.90**	**[1.34–2.70]**	**0.0003**
6p21.33	*C>a*			rec	7/187	14/209	1.79	[0.70–4.54]	0.2214
	Promoter			**dom**	**54/140**	103/120	**2.24**	**[1.49–3.38]**	**0.0001**
*TRAF2*	rs10781522	49.2	38	**add**	**191/197**	**171/279**	**0.64**	**[0.48–0.84]**	**0.0014**
9q34.3	*g>A*			rec	47/147	34/191	0.58	[0.35–0.95]	0.0305
	Intron 9			**dom**	**144/50**	**137/88**	**0.52**	**[0.34–0.79]**	**0.0024**
**APOPTOSIS**									
*PAK2*	rs9325377	49.5	42	add	196/192	190/162	0.72	[0.54–0.94]	0.0173
3q29	*a>G*			**rec**	**55/139**	**36/190**	**0.47**	**[0.29–0.77]**	**0.0023**
	Intron 1			dom	141/53	154/72	0.81	[0.53–1.25]	0.3449
*CD36*	rs4112274	7.51	15.27	**add**	**29/357**	**69/383**	**2.14**	**[1.33–3.44]**	**0.0015**
7q21.11	*C>t*			rec	1/192	5/221	3.92	[0.45–34.2]	0.2169
	Intron 3			**dom**	**28/165**	**64/162**	**2.23**	**[1.34–3.68]**	**0.0018**
**IMMUNOGENIC**									
*EIF2AK3*	rs10167879	17.6	9.9	**add**	**67/313**	**43/393**	**0.48**	**[0.31–0.73]**	**0.0007**
2p11.2	*C>t*			rec	5/185	4/214	0.62	[0.16–2.40]	0.4940
	Intron 14			**dom**	**62/128**	**39/179**	**0.41**	**[0.26–0.66]**	**0.0002**
*SIRPA*	rs6075340	33.5	42.1	add	130/258	191/263	1.50	[1.13–2.00]	0.0055
20p13	*a>G*			**rec**	**17/177**	**47/180**	**2.75**	**[1.51–4.98]**	**0.0009**
	Intron 2			dom	113/81	144/83	1.35	[0.90–2.02]	0.1449
*CD47*	rs12695175	11.4	17.9	**add**	**44/342**	**81/371**	**1.77**	**[1.20–2.63]**	**0.0043**
3q13.12	*T>g*			rec	4/189	10/216	2.23	[0.68–7.30]	0.1850
	Intron 6			**dom**	**40/153**	**71/155**	**1.95**	**[1.23–3.07]**	**0.0041**
**NECROSIS**									
*RAPGEF3*	rs10747521	40.1	36.7	add	155/231	165/285	0.81	[0.61–1.1]	0.1676
12q13.11	*a>G*			**rec**	**36/157**	**23/202**	**0.42**	**[0.23–0.76]**	**0.0040**
	Intron 1			dom	155/231	165/285	1.03	[0.20–0.69]	0.8837
**PARTHANATOS**									
*HK1*	rs7072268	43.0	53.1	**add**	**167/221**	**241/213**	**1.48**	**[1.13–1.94]**	**0.0045**
10q22.1	*t>C*			rec	37/157	70/157	1.57	[1.02–2.42]	0.0412
	Intron 5			dom	130/64	171/56	1.87	[1.18–2.95]	0.0076
**PYROPTOSIS**									
*PRKN*	rs9355950	34.3	22.9	**add**	**133/255**	**104/350**	**0.57**	**[0.42–0.78]**	**0.0004**
6q26	*T>c*			rec	25/169	11/216	0.35	[0.16–0.73]	0.00502
	Intron 4			**dom**	**108/86**	**93/134**	**0.55**	**[0.37–0.81]**	**0.0024**

*Logistic regression association tests were done with allele frequencies (“add”), frequency of homozygotes for the minor allele (“rec”- recessive model), and summed frequencies of heterozygotes and homozygotes for the minor allele (“dom”—dominant model). The minor alleles in our sample are given in lowercase; they are the reference for the associations. In bold, significant associations (p < 0.005). SNP, single nucleotide polymorphism; MAF, minor allele frequency; CONTR, controls; CASE, cases; Model, association tests; OR, odds ratio; CI, confidence interval; TNF, tumor necrosis factor alpha; TRAF2, TNF receptor associated factor 2; PAK2, p21 (RAC1) activated kinase 2; CD36, CD36 molecule; EIF2AK3, eukaryotic translation initiation factor 2 alpha kinase 3 (also known as PERK); SIRPA, signal regulatory protein alpha; CD47, CD47 molecule; RAPGEF3, Rap guanine nucleotide exchange factor 3 (also known as EPAC); HK1, hexokinase 1; PRKN, parkin RBR E3 ubiquitin protein ligase (also known as PARK2)*.

### Functional Annotation *in silico* Analysis

To explore the potential effects of all 10 genetic variants associated with PF, we used functional annotation available in reference public databases. As outlined below, most of the associated variants are associated with gene expression in different tissues (Table 2). Nevertheless, the association of these variants (with disease and with gene expression) may also be explained by strong linkage disequilibrium with other causal variants (hitch-hiking effect).

**Table 2:** Cell death-related gene variants associated with PF.

**Variant**	**Allele associated with PF**	**eQTL effect (25, 29)**	**Tissues**
*TNF_* *rs1800630*	*A* risk	low expression: *TNF* (*p* = 3.71e^−21^) and *LTA* (*p* = 2.88e^−24^) (36). high expression: *DDX39B* (*p* = 2.6e^−4^), *CSNK2B* (*p* = 3.14e^−11^), *HCP5* (*p* = 2.5e^−3^) and *MICB* (*p* = 2.0e^−15^) (36).	Peripheral blood.
*TRAF2_* *rs10781522*	*G* prot	low expression: *C8G* (*p* = 2.6e^−5^), *AGPAT2* (*p* = 9.1e^−4^), and *CLIC3* (*p* = 4.79e^−17^) (32, 36). high expression: *TRAF2* (*p* = 2.73e^−4^), *PHPT1 (p* = 1.2e^−6^)	Skin, peripheral blood, muscle.
*PAK2_* *rs9325377*	*A* prot	low expression: *PAK2* (*p* = 1.2e^−6^) (32). high expression: *PIGX* (*p* = 9.7e^−7^). *PIGX* maps just upstream of *PAK2*, on the same strand of chromosome region 3q29 (32).	Skin, spleen.
*CD36_* *rs4112274*	*T* risk	low expression: *CD36* (*p* = 2.37e^−29^) (36).	Peripheral blood.
*EIF2AK3_* *rs10167879*	*T* prot	low expression: *AC062029.1* (*p* = 3.0e^−4^) (32) high expression: *EIF2AK3* (*p* = 3.5e^−4^), *ANKRD36BP2* (*p* = 1.6e^−6^) (32).	Skin, whole blood.
*SIRPA_* *rs6075340*	*A* risk	low expression: *SIRPA* (*p* = 4.1e^−7^) (32). high expression: *SIRPB1* (*p* = 6.6e^−98^) (32).	Esophagus, skin.
*CD47_* *rs12695175*	*G* risk	low expression: *CD47* (*p* = 3.37e^−4^) (36). high expression: *MYH15* (*p* = 1.3e^−8^) (32).	Peripheral blood, skin.
*RAPGEF3_* *rs10747521*	*A* prot	low expression: *RAPGEF3* (*p* = 2.0 e^−8^) (32). high expression: *PCED1B-AS1* (32).	Lung, heart.
*HK1_* *rs7072268*	*T* risk	high expression: *HK1* (*p* = 2.3 e^−6^) (32).	Tibial nerve.

*PRKN_ rs9355950 is not an eQTL*.

*eQTL, expression quantitative trait loci; LTA, lymphotoxin alpha; DDX39B, dead box polypeptide 39 B; CSNK2B, casein kinase II beta; HCP5, HLA complex P5; MICB, major histocompatibility complex class I-chain related gene B; PHPT1, phosphohistidine phosphatase 1; C8G, complement C8 gamma chain; AGPAT2, 1-acylglycerol-3-phosphate O-acyltransferase 2; CLIC3, chloride intracellular channel 3; PIGX, phosphatidylinositol glycan anchor biosynthesis class X; ANKRD36BP2, ankyrin repeat domain 36B pseudogene 2; SIRPB1, signal regulatory protein beta 1; MYH15, myosin heavy chain 15*.

In accordance with functional annotation, *TNF _rs1800630, TRAF2_rs10781522, PAK2_rs9325377, EIF2AK3_rs10167879, SIRPA_rs6075340, PRKN_rs9355950*, and *HK1_rs7072268* SNPs are located within predicted transcription factor binding sites. On the other hand, the location of *TNF_rs1800630, TRAF2_rs10781522, PAK2_rs9325377, SIRPA_rs6075340, CD47_rs12695175, CD36_rs4112274, HK1_rs7072268*, and *PRKN_rs9355950* SNPs overlap with enhancers and/or promoters that are important in several tissues, including skin, T and B cells from blood (enriched in H3K27Ac) (33, 34). Despite being located downstream of the last exon of *LTA* and far from the transcription start site of *TNF, TNF_rs1800630* is located in a DNase hypersensitive region bound by RNA polymerase II subunit A, providing strong evidence for active transcription of this region in B lymphocytes (33, 34).

Furthermore, the SIRPA protein interacts physically with CD47, suggesting a possible gene interaction/synergistic effect of the associated polymorphisms of both genes on disease susceptibility (35). In fact, carriers of SIRPA_rs6075340*A and CD47_rs12695175*G alleles were more frequent among patients than controls (*OR* = 2.02 [95%CI = 1.08–3.81], *p* = 0.0202). TNF also interacts physically with the TNF receptor TRAF2, as shown in a cervical cancer cell line (35). Thus, an additive susceptibility effect was evident in carriers of TNF_rs1800630*A and TRAF2_rs10781522*A (*OR* = 4.78 [95%CI = 2.18–10.94], *p* < 0.0001) (Supplementary Tables 1, 2).

## Discussion

Although the underlying molecular mechanisms of RCDs overlap considerably, our approach allowed us to suggest the possible role of non-apoptotic RCDs and raise hypotheses to explain the genetic associations that we observed.

### *TNF, TRAF2*, and *PAK2*: Apoptosis and Necroptosis

The genetic associations of TNF_rs1800630*A (also known as −863*A, OR = 1.9, *p* = 0.0003) and TRAF2_rs10781522*G (*OR* = 0.64, *p* = 0.0014) with PF point to a specific role of the death receptor pathway in the disease. As the major proinflammatory cytokine mediating apoptosis and necroptosis, TNF binds TNF type I receptor (TNFRI), activating NF-kB through TRADD (TNFR-associated death domain), and TRAF2 recruitment. This sequence of events culminates in cell survival and inflammation (37, 38). In the absence of TRAF2, TNF binding to TNFRI builds “death-inducing signaling complexes” that can activate either necroptosis or caspase-dependent cell death. In the latter case, this leads to the activation of CASP8 and of the apoptotic cascade (37, 39). On the other hand, TRAF2 was recently reported to positively regulate caspase-2 activation, which initiates apoptosis and is a negative regulator of necroptosis (40, 41). For necroptosis to ensue, caspase inactivity or absence must prevail [thus, in the absence of TRAF2 (38)] (42). Interestingly, although TNF_rs1800630*A carriers present reduced *TNF* expression, TRAF2_rs10781522*G is associated with higher *TRAF2* gene expression in peripheral blood (36). In contrast with *TNF*, the allele from *TRAF2* was associated with PF protection. Since both molecules are known to interact physically (35), certain allelic combinations of these genes present an additive effect toward susceptibility to the disease.

Moreover, many associations of TNF_rs1800630*A with diseases have been reported. Those of enhanced susceptibility to cancer seem to indicate that reduced TNF levels increase the chance of inappropriate cell proliferation, due to insufficient signaling for apoptosis/necroptosis [e.g., hepatocellular carcinoma (43), non-Hodgkin lymphoma (44, 45), gastric cancer (46), and colon cancer (47)]. On the other hand, the same allele was associated with progression to and severity of infections, as for severe malaria, including cerebral malaria (48, 49), HPV-associated oral squamous carcinoma (50), HBV chronification (51), as well as with chronic disorders, which may rely on insufficient immunological response to different kinds of aggressors [steroid-induced osteonecrosis of the femoral head (52), progression of acute pancreatitis to systemic inflammation and multi-organ dysfunction syndrome (53), chronic periodontitis (54), and cardiovascular heart disease (55)].

The TNF_rs1800630*A was associated with predisposition to autoimmune disorders affecting the skin and mucosal tissue, as vitiligo (56), systemic lupus erythematosus (SLE) (57), and Crohn's disease (58). As in these autoimmune skin disorders, the rs1800630*A was associated with susceptibility to endemic PF, in the Brazilian population (*OR* = 1.9, *p* = 0.0003). Interestingly, *TNF* microsatellite polymorphisms were associated with susceptibility to PF in Tunisia, where the disease is also endemic (59). The rs1800630*A causes decreased *TNF* transcription and lower serum TNF levels (51, 60–62). Furthermore, carriers of rs1800629*A (also known as −308*A) presented higher susceptibility to pemphigus (both PV and PF) (63). This allele is associated with TNF expression levels in whole blood, similar as observed for rs1800630*A. Thus, although both occur in different haplotypes (rs1800630_rs1800629*CA and *AG*, with *CG* representing more than 70% of the allelic combinations in the Iberian population), both present the same effect on reducing gene expression and increasing PF susceptibility.

In addition, the *TRAF2* PF protective allele is associated with altered expression of at least five genes implicated in cell survival or death (*PHPT1, PTGDS, LCNL1, C8G, CLIC3*). It is also associated with the expression of one gene related to size and inflammatory itching reaction, after mosquito bites (*AGPAT2*) (64–70). The *AGPAT2* association is particularly interesting, given the epidemiological association of endemic PF with massive and continued exposure to mosquito bites (71, 72).

Another PF susceptibility allele, CD36_rs4112274*T, is associated with decreased *CD36* gene expression in blood. This receptor is a mediator of both endoplasmic reticulum stress and the generation of reactive oxygen species in the intrinsic apoptosis pathway (73). Its predicted down-regulation in most PF patients is an additional argument favoring a less prominent role (if any) for apoptosis in the disease. Its expression in keratinocytes correlates with early wound healing (74). Thus, lower expression is also predicted to increase the extent of PF epidermal lesions.

The PAK2 rs9325377*A/A was associated with PF protection (*OR* = 0.48, *p* = 0.0023). The *PAK2* product is activated through proteolytic cleavage, by caspase-mediated apoptosis. Cleavage of PAK2 regulates morphological changes in apoptotic cells and always correlates with apoptotic cell death (75). The variant PAK2_rs9325377*A occurs in strong linkage disequilibrium with the PAK2_rs6583176*A in the Iberian population (D' = 0.93), and both alleles were associated with increased susceptibility to gastric cancer (76). Thus, it is conceivable that they are associated with apoptosis failure, through yet unknown mechanisms. The association of the PAK2_rs9325377*A with higher *PIGX* expression further reinforces this possibility, since *PIGX* knockdown may inhibit breast cancer cell growth (77).

Taken together, decreased *TNF* gene expression (suggested by TNF_rs1800630*A association) increases PF susceptibility, whereas higher *TRAF2* gene expression (suggested by TRAF2_rs10781522*G association) seems to protect against the disease, as well as PAK2_rs9325377*A/A. Moreover, TRAF2_rs10781522*G is associated with lower expression of *C8G* in the skin, expected to decrease the production of a complement component protecting keratinocytes against apoptosis and necroptosis (32). Higher TNF and TRAF2 levels are thus predicted to be protective against the disease, both preferentially leading to cell survival and inflammation. The fact that we did not find a clear association between any of these gene polymorphisms and apoptosis or necroptosis is in agreement with previous findings using electron microscopy (8, 22).

### *EIF2AK3, CD47*, and *SIRPA*: Immunogenic Cell Death

**Figure 2:**
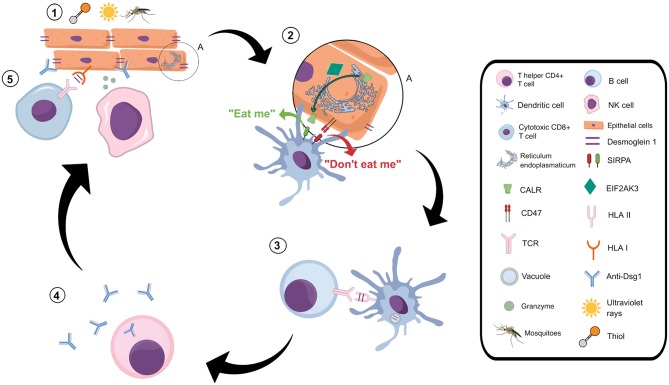
Proposed role of the immunogenic cell death in pemphigus foliaceus. 1: Keratinocytes exposed to exogenous PF risk factors, such as mosquito saliva (71, 72), ultraviolet irradiation (79), and thiol molecules (80), present several stress-derived peptides through HLA class I molecules. These peptides are recognized by cytotoxic T CD8+ cells and natural killer (NK) cells, which launch the immunogenic pathway. 2: This pathway induces damage-associated molecular patterns, including membrane exposure of CALR (81). CALR is transported from the endoplasmic reticulum to the outer cell membrane (81), a process mediated by EIF2AK3 (24). Calreticulin then interacts with SIRPA on the surface of dendritic cells, giving it a signal known as “eat me,” responsible for stimulating phagocytosis (81). Relaying the opposite signal is CD47, an outer surface membrane protein that also interacts with SIRPA and antagonizes the activity of CALR, inhibiting phagocytosis (81). We hypothesize that in PF the “eat me” signals prevail over the “don't eat me” signals, increasing the phagocytosis of keratinocyte debris by dendritic cells. 3: Dendritic cells then present keratinocyte peptides, as those derived from desmoglein 1, to T helper cells. 4: T lymphocytes stimulate auto-antibody production by B lymphocytes. 5: The immunogenic cell death process initiated by exogenous stimuli also activates an adaptive immune response, which includes recruitment, and activation of both cytotoxic T lymphocytes and natural killer cells.

We identified genetic associations of *EIF2AK3*_rs10167879*T with PF protection, and of SIRPA_rs6075340*A/A and CD47_rs12695175*G with susceptibility to PF. These genes encode products that participate in the immunogenic activation by CALR (calreticulin) (78), highlighting CALR as a molecule likely associated with damage-associated molecular patterns (DAMP) that may follow or precede the immunogenic pathway in PF. This cascade can be activated by a relatively limited set of stimuli, that may also include environmental factors associated with PF susceptibility, as UVB (79), thiol and other calcium-sequestering components (80), and components of fly saliva (71, 72) (Figure 2).

Besides its association with PF protection, EIF2AK3_rs10167879*T is also associated with higher *EIF2AK3* and lower *EIF2AK3-DT* (its antisense lncRNA) expression (32). This is the first documented association between this allele and any disease. The association is supported by the fact that deregulation of EIF2AK3 (also known as PERK) has been reported as one of the earliest pathogenic events in PV independently of IgG. Also, EIF2AK3 is activated in keratinocytes exposed to PV serum, as demonstrated by an increase in its phosphorylated levels and in phosphorylation of its target, eIF2α. Decreased EIF2AK3 expression, mediated by small interfering RNA, reduced the effects of PV serum on cell cycle and keratinocyte viability, two PV hallmarks (82). In agreement with this, Cipolla et al. (83) formerly proposed that PF IgG and/or non-IgG extracellular factors may lead to endoplasmic reticulum (ER) stress, resulting in C/EBP-homologous protein (CHOP) induction via EIF2AK3 (PERK), and activation of transcription factor 6 (ATF6). The association with EIF2AK3_rs10167879*T is puzzling, since it would imply that high EIF2AK3 levels are protecting against PF. Nevertheless, it may be explained in the light of its pleiotropy and a possible effect on differential transcription of its two poorly characterized isoforms (31). The SIRPA_rs6075340*A and CD47_rs12695175*G alleles are associated with lower expression of their respective genes (36), in addition to their synergistic association with PF susceptibility. The interaction between CD47 and SIRPA is capable of antagonizing the activity of surface-exposed CALR, responsible for emitting “eat me” signals for phagocytosis (81). The lower expression of those two physically interacting molecules is expected to lead to excessive internalization of cell debris and antigen presentation, increasing PF autoantibody production (Figure 2). This hypothesis agrees with our prior results on complement receptor polymorphisms, which would protect against PF development by modulating scavenging efficiency of acantholytic cell debris (7). A further argument in favor of the importance of the immunogenic cell death pathway in PF is the up-regulated expression of immunogenic deadly granzyme *GZMA* and *GZMB* genes in T lymphocytes of untreated patients with generalized lesions (84).

### *PRKN, HK1*, and *RAPGEF3*: Pyroptosis, Parthanatos and Necrosis

PRKN_rs9355950*C was associated with PF protection (*OR* = 0.57, *p* = 0.0004). This gene encodes the mitophagy-regulating Parkin protein, which prevents cell death by causing ubiquitination of mitochondrial proteins presented by damaged mitochondria. Pyroptosis amplifies mitochondrial damage through caspase 1-driven cleavage and inactivation of PRKN (85). Since the associated effects of this variant on gene expression are unknown, the importance of pyroptosis on PF susceptibility remains elusive.

HK1_rs7072268*A was associated with increased susceptibility to PF (*OR* = 1.48, *p* = 0.0045) and also with higher gene expression levels in nervous tissue (32). Furthermore, this variant is associated with glycated hemoglobin levels (86). It is conceivable that it may be actually associated with enhanced poly(ADP-ribosyl)ation of hexokinase 1, occurring through the activation of PARP [poly(ADP-ribose) polymerase], with consequent glycolysis inhibition and induction of parthanatos-induced cell death (87).

Finally, the RAPGEF3_rs10747521*A/A genotype of the necrosis pathway, associated with protection against the disease (*OR* = 0.42, *p* = 0.004), is also associated with lower *RAPGEF3* gene expression, as well as of its neighboring gene, *SLC18A1*. This protein has a role in inhibiting the p38MAPK pathway (88), activated in PF (83). Higher *PCED1B-AS1* lncRNA expression is also associated with the same variant, predicted to enhance monocyte apoptosis and reduce autophagy (89). The reasons subjacent to this association await the results of future functional studies.

## Conclusion

For the first time, SNPs located within genes involved in all known cell death cascades were systematically investigated in a single disease. The genetic association profile with *TNF, TRAF2, CD36*, and *PAK2* variants favors cell survival and inflammation, instead of apoptosis/necroptosis, to explain resistance against the disease. On the other hand, susceptibility is conferred by variants of *CD47* and *SIRPA* of the immunogenic cell death pathway, proposed to lead to excessive internalization of cell debris, and antigen presentation, which may increase PF autoantibody production. The importance of other pathways as pyroptosis, parthanatos, and necrosis, represented by one association each in our setting, cannot be disclosed and shall be further investigated. Functional validation of these associations, especially of genes encoding common isoforms, as *EIF2AK3*, will provide a better understanding of PF pathogenesis and contribute to the development of new drugs and to therapeutic improvement for the disease.

## Data Availability Statement

The datasets analyzed for this study can be found in the LGMH repository: http://www.lgmh.ufpr.br/data/Supplementary_material_1_Bumiller-Bini_2019.xlsx; http://www.lgmh.ufpr.br/data/Supplementary_material_2_Bumiller-Bini_2019.xlsx.

## Ethics Statement

This study was carried out in accordance with the guidelines of the Conselho Nacional de Ética em Pesquisa (CONEP) with written informed consent from all subjects. All subjects gave written informed consent in accordance with the Declaration of Helsinki. The protocol was approved by CONEP (No. 505.988).

## Author Contributions

MP-E, AB, VB-B, GC, MS, DA, and MB contributed to conception of the work. AB and VB-B designed the study. AB, GC, and MB supervised the study. MP-E provided the samples. MP-E and AB provided resources. DA performed microarray hybridization. VB-B and MS did the statistical analysis and drafted the manuscript. AB, MP-E, GC, DA, and MB edited it. All authors revised the work critically for intellectual content and approved the final version of the work.

### Conflict of Interest

The authors declare that the research was conducted in the absence of any commercial or financial relationships that could be construed as a potential conflict of interest.

## References

[1] Maldonado M Diaz LA Prisayanh P Yang J Qaqish BF Aoki V. Divergent specificity development of IgG1 and IgG4 autoantibodies in endemic pemphigus foliaceus (fogo selvagem). ImmunoHorizons. (2017) 1:71–80. 10.4049/immunohorizons.1700029 28868524 PMC5577939

[2] Evangelista F Roth AJ Prisayanh P Temple BR Li N Qian Y. Pathogenic IgG4 autoantibodies from endemic pemphigus foliaceus recognize a desmoglein-1 conformational epitope. J. Autoimmun. (2018) 89:171–85. 10.1016/j.jaut.2017.12.017 29307589 PMC5902409

[3] Spindler V Drenckhahn D Zillikens D Waschke J. Pemphigus IgG causes skin splitting in the presence of both desmoglein 1 and desmoglein 3. Am J Pathol. (2007) 171:906–16. 10.2353/ajpath.2007.070028 17640963 PMC1959479

[4] Bystryn JC Rudolph JL. Pemphigus. Lancet. (2005) 366:61–73. 10.1016/S0140-6736(05)66829-8 15993235

[5] Hans-Filho G Dos Santos V Katayama JH Aoki V Rivitti EA Sampaio SAP. An active focus of high prevalence of Fogo selvagem on an Amerindian reservation in Brazil. J Invest Dermatol. (1996) 107:68–75. 10.1111/1523-1747.ep12298213 8752842

[6] Diaz LA Sampaio SA Rivitti EA Martins CR Cunha PR Lombardi C. Endemic pemphigus foliaceus (Fogo Selvagem): I. Clinical features and immunopathology. J Am Acad Dermatol. (1989) 20:657–69. 10.1016/S0190-9622(89)70079-7 2654208

[7] Bumiller-Bini V Cipolla GA Almeida RC Petzl-Erler ML Augusto DG Boldt ABW. Sparking fire under the skin? Answers from the association of complement genes with pemphigus foliaceus. Front Immunol. (2018) 9:695. 10.3389/fimmu.2018.00695 29686679 PMC5900433

[8] Janse IC Van Der Wier G Jonkman MF Pas HH Diercks GF. No evidence of apoptotic cells in pemphigus acantholysis. J Invest Dermatol. (2014) 134:2039–41. 10.1038/jid.2014.60 24487306

[9] Gniadecki R Jemec GB Thomsen BM Hansen M. Relationship between keratinocyte adhesion and death: anoikis in acantholytic diseases. Arch Dermatol Res. (1998) 290:528–32. 10.1007/s004030050347 9836502

[10] Puviani M Marconi A Cozzani E Pincelli C. Fas ligand in pemphigus sera induces keratinocyte apoptosis through the activation of caspase-8. J Invest Dermatol. (2003) 120:164–7. 10.1046/j.1523-1747.2003.12014.x 12535213

[11] Wang X Brégégère F Frusić ZM Feinmesser M Michel B Milner Y. Possible apoptotic mechanism in epidermal cell acantholysis induced by pemphigus vulgaris autoimmunoglobulins. Apoptosis. (2004) 9:131–43. 10.1023/B:APPT.0000018795.05766.1f 15004510

[12] Cuevas-Gonzalez JC Vega-Memíje ME García-Vázquez FJ Aguilar-Urbano MA. Detection of apoptosis in pemphigus vulgaris by TUNEL technique. An Bras Dermatol. (2016) 91:296–9. 10.1590/abd1806-4841.20164598 27438195 PMC4938272

[13] Frusic-Zlotkin M Raichenberg D Wang X David M Michael B Milner Y. Apoptotic mechanism in pemphigus autoimmunoglobulins-induced acantholysis–possible involvement of the EGF receptor. Autoimmunity. (2006) 39:563–75. 10.1080/08916930600971836 17101500

[14] Li N Zhao M Wang J Liu Z Diaz LA. Involvement of the apoptotic mechanism in pemphigus foliaceus autoimmune injury of the skin. J Immunol. (2009) 182:711–7. 10.4049/jimmunol.182.1.711 19109205 PMC2716799

[15] Deyhimi P Alishahi B. Study of extrinsic apoptotic pathway in oral pemphigus vulgaris using TNFR 1 and FasL immunohistochemical markers and TUNEL technique. J Dent. (2018) 19:132–41. 29854887 PMC5960733

[16] Schmidt E Waschke J. Apoptosis in pemphigus. Autoimmun Rev. (2009) 8:533–7. 10.1016/j.autrev.2009.01.011 19189866

[17] Schmidt E Gutberlet J Siegmund D Berg D Wajant H Waschke J Apoptosis is not required for acantholysis in pemphigus vulgaris. Am J Physiol Cell Physiol. (2009) 296:C162–72. 10.1152/ajpcell.00161.2008 18987254

[18] Lee HE Berkowitz P Jolly PS Diaz LA Chua MP Rubenstein DS. Biphasic activation of p38MAPK suggests that apoptosis is a downstream event in pemphigus acantholysis. J Biol Chem. (2009) 284:12524–32. 10.1074/jbc.M808204200 19270308 PMC2673318

[19] Rodrigues DBR Pereira SAL Reis MA Adad SJ Caixeta JE Chiba AM. *In situ* detection of inflammatory cytokines and apoptosis in pemphigus foliaceus patients. Arch Pathol Lab Med. (2009) 133:97–100. 10.1043/1543-2165-133.1.97 19123745

[20] Dusek RL Getsios S Chen F Park JK Amargo EV Cryns VL. The differentiation-dependent desmosomal cadherin desmoglein 1 is a novel caspase-3 target that regulates apoptosis in keratinocytes. J Biol Chem. (2006) 281:3614–24. 10.1074/jbc.M508258200 16286477

[21] Zuccolotto I Roselino AM Ramalho LNZ Zucoloto S. Apoptosis and p63 expression in the pathogenesis of bullous lesions of endemic pemphigus foliaceus. Arch Dermatol Res. (2003) 295:284–6. 10.1007/s00403-003-0434-3 14598177

[22] Sokol E Kramer D Diercks GF Kuipers J Jonkman MF Pas HH. Large-scale electron microscopy maps of patient skin and mucosa provide insight into pathogenesis of blistering diseases. J Invest Dermatol. (2015) 135:1763–70. 10.1038/jid.2015.109 25789704

[23] Lotti R Shu E Petrachi T Marconi A Palazzo E Quadri M. Soluble fas ligand is essential for blister formation in pemphigus. Front Immunol. (2018) 9:370. 10.3389/fimmu.2018.00370 29535737 PMC5834757

[24] Galluzzi L Vitale I Aaronson SA Abrams JM Adam D Agostinis. Molecular mechanisms of cell death: recommendations of the nomenclature committee on cell death 2018. Cell Death Differ. (2018) 25:486–541. 10.1038/s41418-017-0012-4 29362479 PMC5864239

[25] Galluzzi L Bravo-San Pedro JM Kepp O Kroemer G. Regulated cell death and adaptive stress responses. Cell Mol Life Sci. (2016) 73:2405–10. 10.1007/s00018-016-2209-y 27048813 PMC11108439

[26] Sambrook J Russell DW Molecular Cloning: A Laboratory Manual. New York, NY: CSHL Press (2001).

[27] Benjamin DJ Berger JO Johannesson M Nosek BA Wagenmakers EJ Berk R. Redefine statistical significance. Nat Hum Behav. (2018) 2:6–10. 10.1038/s41562-017-0189-z 30980045

[28] Purcell S Neale B Todd-Brown K Thomas L Ferreira MAR Bender D. PLINK: a tool set for whole-genome association and population-based linkage analyses. Am J Hum Genet. (2007) 81:559–75. 10.1086/519795 17701901 PMC1950838

[29] Agresti A A survey of exact inference for contingency tables. Stat Sci. (1992):131–53. 10.1214/ss/1177011454

[30] Machiela MJ Chanock SJ. LDlink: a web-based application for exploring population-specific haplotype structure and linking correlated alleles of possible functional variants. Bioinformatics. (2015) 31:3555–7. 10.1093/bioinformatics/btv402 26139635 PMC4626747

[31] Hunt SE McLaren W Gil L Thormann A Schuilenburg H Sheppard D. Ensembl variation resources. Database. (2018) 2018:bay119. 10.1093/database/bay119 30576484 PMC6310513

[32] Consortium TG The genotype-tissue expression (GTEx) project. Nat Genet. (2013) 45:580–5. 10.1038/ng.2653 23715323 PMC4010069

[33] Kent WJ Sugnet CW Furey TS Roskin KM Pringle TH Zahler AM. The human genome browser at UCSC. Genome Res. (2002) 12:996–1006. 10.1101/gr.229102 12045153 PMC186604

[34] Ward LD Kellis M. HaploReg: a resource for exploring chromatin states, conservation, and regulatory motif alterations within sets of genetically linked variants. Nucleic Acids Res. (2012) 40:D930–4. 10.1093/nar/gkr917 22064851 PMC3245002

[35] Breuer K Foroushani AK Laird MR Chen C Sribnaia A Lo R. InnateDB: systems biology of innate immunity and beyond-recent updates and continuing curation. Nucleic Acids Res. (2012) 41:D1228–33. 10.1093/nar/gks1147 23180781 PMC3531080

[36] Westra HJ Peters MJ EskoT Yaghootkar H Schurmann C Kettunen J. Systematic identification of trans eQTLs as putative drivers of known disease associations. Nat Genet. (2013) 45:1238–43. 10.1038/ng.2756 24013639 PMC3991562

[37] Borghi A Verstrepen L Beyaert R TRAF2 multitasking in TNF receptor-induced signaling to NF-κB, MAP kinases and cell death. Biochem Pharmacol. (2016) 15:1–10. 10.1016/j.bcp.2016.03.009 26993379

[38] Hanson B. Necroptosis: a new way of dying? Cancer Biol Ther. (2016) 17:899–910. 10.1080/15384047.2016.1210732 27434654 PMC5036404

[39] Gonzalvez F Lawrence D Yang B Yee S Pitti R Marsters S. TRAF2 Sets a threshold for extrinsic apoptosis by tagging caspase-8 with a ubiquitin shutoff timer. Mol Cell. (2012) 48:888–99. 10.1016/j.molcel.2012.09.031 23142077

[40] Robeson AC Lindblom KR Wojton J Kornbluth S Matsuura K. Dimer-specific immunoprecipitation of active caspase-2 identifies TRAF proteins as novel activators. EMBO J. (2018) 37:e97072. 10.15252/embj.201797072 29875129 PMC6043850

[41] Zamaraev AV Kopeina GS Buchbinder JH Zhivotovsky B Lavrik IN. Caspase-2 is a negative regulator of necroptosis. Int J Biochem Cell Biol. (2018) 102:101–8. 10.1016/j.biocel.2018.07.006 30025878

[42] Berghe TV Vanlangenakker N Parthoens E Deckers W Devos M Necroptosis, necrosis and secondary necrosis converge on similar cellular disintegration features. Cell Death Differ. (2010) 17:922–30. 10.1038/cdd.2009.184 20010783

[43] Yang Y Qiu XQ Yu HP Zeng XY Bei CH. TNF−863 polymorphisms and the risk of hepatocellular carcinoma. Exp Therm Med. (2012) 3:513–8. 10.3892/etm.2011.418 22969921 PMC3438725

[44] Ye X Zhao K Wu C Hu P Fu H. Associations between genetic variants in immunoregulatory genes and risk of non-Hodgkin lymphoma in a Chinese population. Oncotarget. (2017) 8:10450–7. 10.18632/oncotarget.14426 28060727 PMC5354671

[45] Zhang J Ye X Wu C Fu H Xu W Hu P. Modeling gene-environment interaction for the risk of non-hodgkin lymphoma. Front Oncol. (2019) 8:657. 10.3389/fonc.2018.00657 30693270 PMC6340069

[46] Sultana Z Bankura B Pattanayak AK Sengupta D Sengupta M Saha ML. Association of Interleukin-1 beta and tumor necrosis factor-alpha genetic polymorphisms with gastric cancer in India. Environ Mol Mutagen. (2018) 59:653–67. 10.1002/em.22208 30094865

[47] Slattery ML Lundgreen A Bondurant KL Wolff RK. Tumor necrosis factor-related genes and colon and rectal cancer. Int J Mol Epidemiol Genet. (2011) 2:328–38. 22199996 PMC3243449

[48] Sinha S Mishra SK Sharma S Patibandla PK Mallick PK Sharma SK. Polymorphisms of TNF-enhancer and gene for FcγRIIa correlate with the severity of falciparum malaria in the ethnically diverse Indian population. Malaria J. (2008) 7:13. 10.1186/1475-2875-7-13 18194515 PMC2245971

[49] Hananantachai H Patarapotikul J Ohashi J Naka I Krudsood S Looareesuwan S. Significant association between TNF-α (TNF) promoter allele (– 1031C,– 863C, and– 857C) and cerebral malaria in Thailand. Tissue Antigens. (2007) 69:277–80. 10.1111/j.1399-0039.2006.00756.x 17493155

[50] Jin L Sturgis EM Zhang Y Huang Z Song X Li C. Association of tumor necrosis factor-alpha promoter variants with risk of HPV-associated oral squamous cell carcinoma. Mol Cancer. (2013) 12:80. 10.1186/1476-4598-12-80 23870134 PMC3725173

[51] Kao PC Wu JF Ni YH Lin YT Chen HL Huey-Jen SH. Tumour necrosis factor-α promoter region polymorphisms affect the course of spontaneous HBsAg clearance. Liver Int. (2010) 30:1448–53. 10.1111/j.1478-3231.2010.02340.x 20825556

[52] Liu Y Jiang W Liu S Su X Zhou S. Combined effect of TNF polymorphisms and hypoxia on steroid-induced osteonecrosis of femoral head. Int J Clin Exp Pathol. (2015) 8:3215–9. 26045843 PMC4440152

[53] Bishehsari F Sharma A Stello K Toth C O'Connell MR Evans AC TNA alfa gene (TNFA) variants increase risk for multi-organ dysfunction syndrome (MODS) in acute pancreatitis. Pancreatology. (2012) 12:113–8. 10.1016/j.pan.2012.02.014 22487520 PMC4350817

[54] Ding C Ji X Chen X Xu Y Zhong L. TNF-α gene promoter polymorphisms contribute to periodontitis susceptibility: evidence from 46 studies. J Clin Periodontol. (2014) 41:748–59. 10.1111/jcpe.12279 24905365

[55] Hernández-Díaz Y Tovilla-Zárate CA Juárez-Rojop I Baños-González MA Torres-Hernández ME López-Narváez ML. The role of gene variants of the inflammatory markers CRP and TNF in cardiovascular heart disease: systematic review and meta-analysis. Int J Clin Exp Med. (2015) 8:11958–84. 26550110 PMC4612795

[56] Laddha NC Dwivedi M Begum R. Increased Tumor Necrosis Factor (TNF)-α and its promoter polymorphisms correlate with disease progression and higher susceptibility towards vitiligo. PLoS ONE. (2012) 7:e52298. 10.1371/journal.pone.0052298 23284977 PMC3527546

[57] Solus JF Chung CP Oeser A Li C Rho YH Bradley KM. Genetics of serum concentration of IL-6 and TNFα in systemic lupus erythematosus and rheumatoid arthritis: a candidate gene analysis. Clin Rheumatol. (2015) 34:1375–82. 10.1007/s10067-015-2881-6 25652333 PMC4526456

[58] Negoro K Kinouchi Y Hiwatashi N Takahashi S Takagi S Satoh J. Crohn's disease is associated with novel polymorphisms in the 5'-flanking region of the tumor necrosis factor gene. Gastroenterology. (1999) 117:1062–8. 10.1016/S0016-5085(99)70390-2 10535868

[59] Abida O Mahfoudh N Kammoun A Gaddour L Hakim F Toumi A. Polymorphisms of HLA microsatellite marker in Tunisian pemphigus foliaceus. Hum Immunol. (2013) 74:104–9. 10.1016/j.humimm.2012.10.013 23073295

[60] Skoog T Hooft FM Kallin B Jovinge S Boquist S Nilsson J. A common functional polymorphism (C → A substitution at position– 863) in the promoter region of the tumour necrosis factor-α (TNF) gene associated with reduced circulating levels of TNF. Hum Mol Genet. (1999) 8:1443–9. 10.1093/hmg/8.8.1443 10400991

[61] Boehm J Hauner K Grammer J Dietrich W Wagenpfeil S Braun S. Tumor necrosis factor-α-863 C/A promoter polymorphism affects the inflammatory response after cardiac surgery. Eur J Cardio-Thor Surg. (2011) 40:e50–4. 10.1016/j.ejcts.2011.01.084 21450487

[62] Gupta S A decision between life and death during TNF-induced signaling. J Clin Immunol. (2002) 22:185–94. 10.1023/A:1016089607548 12148593

[63] Mosaad YM Fathy H Fawzy Z El-Saied MA. Tumor necrosis factor-α−308 G>A and interleukin-6−174 G>C promoter polymorphisms and pemphigus. Hum Immunol. (2012) 73:560–5. 10.1016/j.humimm.2012.02.001 22365967

[64] Liu X Wang J Chen M Liu S Yu X Wen F. Combining data from TCGA and GEO databases and reverse transcription quantitative PCR validation to identify gene prognostic markers in lung cancer. OncoTargets Ther. (2019) 12:709–20. 10.2147/OTT.S183944 30718962 PMC6345189

[65] Tichy A Kabacik S O'Brien G Pejchal J Sinkorova Z Kmochova A. The first *in vivo* multiparametric comparison of different radiation exposure biomarkers in human blood. PLoS ONE. (2018) 13:e0193412. 10.1371/journal.pone.0193412 29474504 PMC5825084

[66] Wu CC Shyu RY Wang CH Tsai TC Wang LK Chen ML. Involvement of the prostaglandin D2 signal pathway in retinoid-inducible gene 1 (RIG1)-mediated suppression of cell invasion in testis cancer cells. Biochim Biophys Acta Mol Cell Res. (2012) 1823:2227–36. 10.1016/j.bbamcr.2012.08.013 22960220

[67] Omori K Morikawa T Kunita A Nakamura T Aritake K Urade Y. Lipocalin-type prostaglandin D synthase-derived PGD2 attenuates malignant properties of tumor endothelial cells. T J Pat. (2018) 244:84–96. 10.1002/path.4993 29124765

[68] Kehrer JP. Lipocalin-2: pro-or anti-apoptotic? Cel Biol Tox. (2010) 26:83–9. 10.1007/s10565-009-9119-9 19160065

[69] Shi H Williams JA Guo L Stampoulis D Cordeiro MF Moss SE Exposure to the complement C5b-9 complex sensitizes W photoreceptor cells to both apoptosis and necroptosis. Apoptosis. (2015) 20:433–43. 10.1007/s10495-015-1091-7 25735751 PMC4348505

[70] Jones AV Tilley M Gutteridge A Hyde C Nagle M Ziemek D. GWAS of self-reported mosquito bite size, itch intensity and attractiveness to mosquitoes implicates immune-related predisposition loci. Hum Mol Gen. (2017) 26:1391–406. 10.1093/hmg/ddx036 28199695 PMC5390679

[71] Lombardi C Borges PC Chaul A Sampaio SA Rivitti EA Friedman H. Environmental risk factors in the endemic pemphigus foliaceus (fogo selvagem). J Invest Dermatol. (1992) 98:847–50. 10.1111/1523-1747.ep12456932 1593148

[72] Vernal S Pepinelli M Casanova C Goulart TM Kim O De Paula NA. Insights into the epidemiological link between biting flies and pemphigus foliaceus in Southeastern Brazil. Acta Trop. (2017) 176:455–62. 10.1016/j.actatropica.2017.09.015 28941730

[73] Kim DH Cho YM Lee KH Jeong SW Kwon OJ. Oleate protects macrophages from palmitate-induced apoptosis through the downregulation of CD36 expression. Biochem Biophy Res Com. (2017) 488:477–82. 10.1016/j.bbrc.2017.05.066 28522296

[74] Simon M Jr Juhász I Herlyn M Hunyadi J. Thrombospondin receptor (CD36) expression of human keratinocytes during wound healing in a SCID mouse/human skin repair model. J Dermatol. (1996) 23:305–9. 10.1111/j.1346-8138.1996.tb04020.x 8675818

[75] Rudel T Bokoch GM. Membrane and morphological changes in apoptotic cells regulated by caspase-mediated activation of PAK2. Science. (1997) 276:1571–4. 10.1126/science.276.5318.1571 9171063

[76] Hyland PL Lin SW Hu N Zhang H Wang L Su H. Genetic variants in fas signaling pathway genes and risk of gastric cancer. Int J Cancer. (2014) 134:822–3. 10.1002/ijc.28415 23921907 PMC3858487

[77] Nakakido M Tamura K Chung S Ueda K Fujii R Kiyotani K. Phosphatidylinositol glycan anchor biosynthesis,class X containing complex promotes cancer cell proliferation through suppression of EHD2 and ZIC1, putative tumor suppressors. Int J Oncol. (2016) 49:868–76. 10.3892/ijo.2016.3607 27572108 PMC4948962

[78] Kepp O Senovilla L Vitale I Vacchelli E Adjemian S Agostinis P. Consensus guidelines for the detection of immunogenic cell death. Oncoimmunol. (2014) 3:e955691. 10.4161/21624011.2014.955691 25941621 PMC4292729

[79] Reis VM Toledo RP Lopez A Diaz LA Martins JE. UVB-induced acantholy-sis in endemic pemphigus foliaceus (fogo selvagem) and pemphigus vulgaris. J Am Acad Dermatol. (2000) 42:571–6. 10.1016/S0190-9622(00)90167-1 10727300

[80] Abréu-vélez AM Messias-Reason IJ Howard MS Roselino A. Endemic pemphigus foliaceus over a century: part I. N Am J Med Sci. (2010) 2:51–9. 22624115 PMC3354435

[81] Barclay AN Van den Berg TK. The interaction between signal regulatory protein alpha (SIRPα) and CD47: structure, function, and therapeutic target. Ann Rev Immunol. (2014) 32:25–50. 10.1146/annurev-immunol-032713-120142 24215318

[82] Lanza A Lanza M Santoro R Soro V Prime SS Cirillo N. Deregulation of PERK in the autoimmune disease pemphigus vulgaris occurs via IgG-independent mechanisms. Brit J Dermatol. (2011) 164:336–43. 10.1111/j.1365-2133.2010.10084.x 21039404

[83] Cipolla GA Park JK Lavker RM Petzl-Erler ML. Crosstalk between signaling pathways in pemphigus: a role for endoplasmic reticulum stress in p38 mitogen-activated protein kinase activation? Front Immunol. (2017) 8:1022. 10.3389/fimmu.2017.01022 28928733 PMC5591886

[84] Malheiros D Panepucci RA Roselino AM Araújo AG Zago MA Petzl-Erler ML. Genome-wide gene expression profiling reveals unsuspected molecular alterations in pemphigus foliaceus. Immunology. (2014) 143:381–95. 10.1111/imm.12315 24813052 PMC4212952

[85] Yu J Nagasu H Murakami T Hoang H Broderick L Hoffman HM. Inflammasome activation leads to Caspase-1–dependent mitochondrial damage and block of mitophagy. Proc Natl Acad Sci USA. (2014) 111:15514–9. 10.1073/pnas.1414859111 25313054 PMC4217429

[86] Paré G Chasman DI Parker AN Nathan DM Miletich JP Zee RY. Novel association of HK1 with glycated hemoglobin in a non-diabetic population: a genome-wide evaluation of 14,618 participants in the Women's Genome Health Study. PLoS Genet. (2008) 4:e1000312. 10.1371/journal.pgen.1000312 19096518 PMC2596965

[87] Song H Yoon SP Kim J. Poly (ADP-ribose) polymerase regulates glycolytic activity in kidney proximal tubule epithelial cells. Anat Cel Biol. (2016) 49:79–87. 10.5115/acb.2016.49.2.79 27382509 PMC4927434

[88] Curtiss E Jiang Y Liu L Hawthorne C Zhang J Steinle JJ. Epac1 restores normal insulin signaling through a reduction in inflammatory cytokines. Med Inflamm. (2018) 2018:3809092. 10.1155/2018/3809092 30116147 PMC6079497

[89] Li M Cui J Niu W Huang J Feng T Sun B. Long non-coding PCED1B-AS1 regulates macrophage apoptosis and autophagy by sponging miR-155 in active tuberculosis. Biochem Biophys Res Commun. (2019) 509:803–9. 10.1016/j.bbrc.2019.01.005 30621915

