# Supplementary material for: Condemned or Not to Die? Gene Polymorphisms Associated With Cell Death in Pemphigus Foliaceus
Source: Front Immunol. 2019 Oct 18;10:2416. doi: 10.3389/fimmu.2019.02416 (PMC6813369; doi:10.3389/fimmu.2019.02416)
Supplement: Supplementary file 1 [file Data_Sheet_1.pdf]

Supplementary Material

Table 1: Stratified analyses between rs6075340 (*A/G*) of *SIRPA* and rs12695175 *(T/G)* of *CD47*.

	SIRPA	*CD47*	Cases	Controls	OR	[95%CI]	p
	rs6075340	rs12695175					
A)			n = 88	n = 47			
	A/A	*T/T*	32	12	1.66	[0.71 – 4.03]	0.2489
	G+	*G+*	56	35	0.60	[0.24 – 1.39]	
**B)**			**n = 138**	**n = 146**			
	A/A	***G+***	**15**	**5**	**3.42**	**[1.14 – 12.39]**	**0.0190**
	G+	***T/T***	**123**	**141**	**0.29**	**[0.08 – 0.87]**	
C)			n = 173	n = 172			
	A/A	*G/G*	2	0	-	-	0.4986
	G+	*T+*	171	172	-	-	
D)			n = 53	n = 21			
	A/A	*T+*	45	17	1.31	[0.25 – 5.74]	0.7314
	G+	*G/G*	8	4	0.75	[0.17 – 3.89]	
E)			n = 117	n = 99			
	A+	*T/T*	95	86	0.65	[0.28 – 1.45]	0.2734
	G/G	*G+*	22	13	1.52	[0.68 – 3.52]	
**F)**			**n = 109**	**n = 94**			
	A+	***G+***	**49**	**27**	**2.02**	**[1.08 – 3.81]**	**0.0202**
	G/G	***T/T***	**60**	**67**	**0.49**	**[0.26 – 0.92]**	
G)			n = 88	n = 82			
	A+	*G/G*	8	3	2.61	[0.60 – 15.88]	0.2144
	G/G	*T+*	80	79	0.38	[0.06 - 1.66]	
H)			n = 138	n = 111			
	A+	*T+*	136	110	0.61	[0.01 – 12.04]	1.0
	G/G	*G/G*	2	1	1.61	[0.08 – 96.23]	

In bold: significant (Fisher exact test). Fisher exact test was performed using the online web statistical calculator Astasa [www.astatsa.com]. OR: Odds Ratio. CI: Confidence interval. SNP: single nucleotide polymorphisms.

Table 2: Stratified analyses between 10781522 (*A/G*) of *TRAF2* and rs1800630 *(A/C)* of *TNF*.

	TRAF2	*TNF*	Cases	Controls	OR	[95%CI]	p
	rs10781522	rs1800630					
A)			n = 129	n = 145			
	A/A	*A/A*	4	4	1.12	[0.20 – 6.18]	
	G+	*C+*	125	141	0.88	[0.16 – 4.86]	1.0
B)			n = 94	n = 49			
	A/A	*C+*	84	46	0.54	[0.09 – 2.28]	
	G+	*A/A*	10	3	1.81	[0.43 – 10.79]	0.5428
C)			n = 115	n = 80			
	A/A	*C/C*	50	38	0.85	[0.46 – 1.57]	
	G+	*A+*	65	42	1.17	[0.63 – 2.17]	0.6610
**D)**			**n = 108**	**n = 114**			
	A/A	***A+***	**38**	**12**	**4.58**	**[2.16 – 10.34]**	
	G+	***C/C***	**70**	**102**	**0.21**	**[0.09 – 0.46]**	**<0.0001**
E)			n = 42	n = 52			
	A+	*A/A*	11	6	2.69	[0.81 – 9.84]	
	G/G	*C+*	31	46	0.37	[0.10 – 1.23]	0.1044
F)			n = 181	n = 142			
	A+	*C+*	178	141	0.42	[0.01 – 5.31]	
	G/G	*A/A*	3	1	2.37	[0.18 – 125.54]	0.6336
G)			n = 126	n = 125			
	A+	*C/C*	106	109	0.77	[0.35 – 1.67]	
	G/G	*A+*	20	16	1.28	[0.59 – 2.80]	0.5896
**H)**			**n = 97**	**n = 69**			
	A+	***A+***	**83**	**38**	**4.78**	**[2.18 – 10.94]**	
	G/G	***C/C***	**14**	**31**	**0.20**	**[0.09 – 0.45]**	**<0.0001**

In bold: significant (Fisher exact test). Fisher exact test was performed using the online web statistical calculator Astasa [www.astatsa.com]. OR: Odds Ratio. CI: Confidence interval. SNP: single nucleotide polymorphisms.
